# Prediction of driving behavior based on emotional intelligence & driving risk perception

**Published:** 2019-08

**Authors:** Masoumeh Afsari, Alireza Moghisi

**Affiliations:** ^*a*^Center of non-communicable disease management- Ministry of Health, Iran.

## Abstract

**Background:**

The aim of this study was to predict driving behavior based on emotional intelligence & driving risk perception.

**Methods:**

This research was descriptive & correlational. The sample size consisted of 400 drivers from 5 taxi terminals of different areas of Tehran megacity by convenience sampling. The tools which used in this study were as follows: Manchester driving behavior questionnaire, Ulleberg & Rundmo driving risk perception questionnaire, Shrink emotional intelligence questionnaire. Data analysis was based on Pearson correlation & multiple simultaneous regression.

**Results:**

Analysis revealed that the relationship between driving behavior & driving risk perception was significant & negative (r = -0/34) (ɑ = 0/01). The relationship between driving behavior & emotional intelligence was significant & negative (r = -0/28) (ɑ = 0/01).

**Conclusions:**

Emotional intelligence & driving risk perception could predict 16% of driving behavior. That means by increasing emotional intelligence & promote driving risk perception among taxi drivers, the risky driving behavior could be decreased. In the other words drivers who do risky driving behavior and commit driving offenses, their emotional intelligence is less than other drivers.

**Keywords:**

Risky driving behavior, Driving risk perception, Emotional intelligence

